# Progress in ZnO Nanosensors

**DOI:** 10.3390/s21165502

**Published:** 2021-08-16

**Authors:** Miaoling Que, Chong Lin, Jiawei Sun, Lixiang Chen, Xiaohong Sun, Yunfei Sun

**Affiliations:** 1 College of Electronic and Information Engineering, Suzhou University of Science and Technology, Suzhou 215009, China; quemiaoling@126.com (M.Q.); tayloree2009@163.com (J.S.); chenlixiang0426@163.com (L.C.); zixuan19861002@126.com (X.S.); 2 Jiangxi Province Key Laboratory of Polymer Micro/Nano Manufacturing and Devices, School of Chemistry, Biology and Materials Science, East China University of Technology, Nanchang 330013, China; lin_chong@ecut.edu.cn

**Keywords:** ZnO nanostructure, synthetic method, pressure sensor, gas sensor, photoelectric sensor, biosensor, temperature sensor

## Abstract

Developing various nanosensors with superior performance for accurate and sensitive detection of some physical signals is essential for advances in electronic systems. Zinc oxide (ZnO) is a unique semiconductor material with wide bandgap (3.37 eV) and high exciton binding energy (60 meV) at room temperature. ZnO nanostructures have been investigated extensively for possible use as high-performance sensors, due to their excellent optical, piezoelectric and electrochemical properties, as well as the large surface area. In this review, we primarily introduce the morphology and major synthetic methods of ZnO nanomaterials, with a brief discussion of the advantages and weaknesses of each method. Then, we mainly focus on the recent progress in ZnO nanosensors according to the functional classification, including pressure sensor, gas sensor, photoelectric sensor, biosensor and temperature sensor. We provide a comprehensive analysis of the research status and constraints for the development of ZnO nanosensor in each category. Finally, the challenges and future research directions of nanosensors based on ZnO are prospected and summarized. It is of profound significance to research ZnO nanosensors in depth, which will promote the development of artificial intelligence, medical and health, as well as industrial, production.

## 1. Introduction

Sensors, as devices that can sense and convert various measured signals, have been developed and improved upon for over a decade [1]. Due to the small size, high sensitivity and high precision, nanosensors have attracted extensive attention in recent years [2,3] and they are used in numerous fields, such as healthcare, military, industrial control and robotics, networking and communications and environmental monitoring.

ZnO, with a wide bandgap and high exciton binding energy, has long been investigated for a variety of applications, such as sensors, light-emitting diodes, solar cells and laser diodes, which can work in the wavelength ranging from ultraviolet to the visible region [4,5]. Compared with bulk materials, ZnO nanostructures exhibit many special properties, such as quantum size effect, high specific surface area and dimension limitation effect. Using the excellent characteristics of ZnO nanomaterials, various sensors, piezoelectric and photoelectric devices have been prepared [6,7], which are widely used in biotechnology, scientific research and industrial production. ZnO nanosensors have aroused extensive concern and researchers are working to achieve nanosensors with advantages such as low cost, high stability, high sensitivity, fast response and fast recovery [8,9]. Here, we provide an overview of recent progress in ZnO nanosensors. We concentrate on the ZnO nanostructures and their fabrication methods; then, we give a review of ZnO nanosensors by classification of their functions, respectively.

## 2. ZnO Nanostructures and Synthetic Method

### 2.1. ZnO Nanostructures

**Figure 1:**
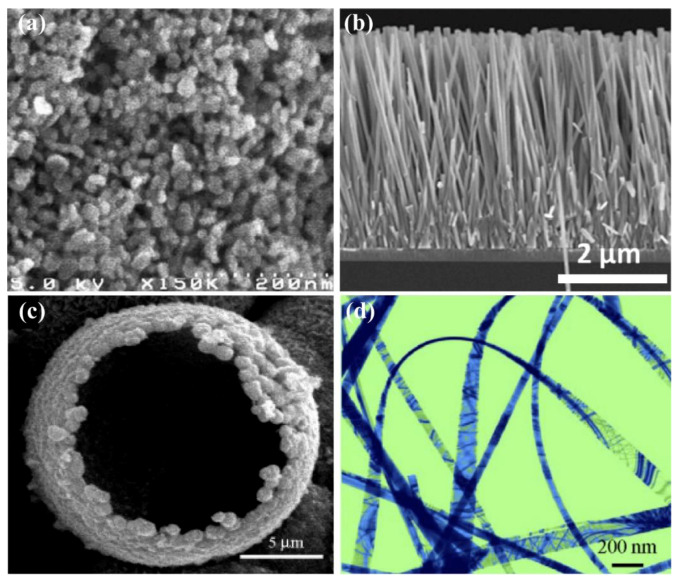
SEM images of ZnO nanostructures. (**a**) Nanoparticle. Reproduced with permission [12]. Copyright 2007, Elsevier. (**b**) Nanowire. Reproduced with permission [13]. Copyright 2014, Royal Society of Chemistry. (**c**) Hollow sphere. Reproduced with permission [21]. Copyright 2006, American Chemical Society. (**d**) Nanobelt. Reproduced with permission [22]. Copyright 2009, Elsevier.

ZnO, as an important semiconducting material, exhibits splendid and abundant nanostructures [10]. According to the size and morphology, ZnO nanomaterials can be divided into four categories, including zero-dimensional nanomaterials, such as quantum dot (QD) [11] and nanoparticle (NP) [12], one-dimensional nanomaterials, such as nanowire (NW) [13], nanobelt (NB) [14], nanoneedle (NN) [15] and nanorod (NR) [16], two-dimensional nanomaterials, such as nanofilm [17] and nanosheet (NS) [18] and three-dimensional nanomaterials, such as porous ZnO materials [19] and nanocluster [20]. Figure 1 shows the scanning electron microscope (SEM) images of some ZnO nanostructures.

These four categories of ZnO nanostructures tend to have different characteristics. Zero-dimensional ZnO nanomaterials have an obvious quantum limiting effect and the energy gap increases up to 4.5 eV with the particle size decrease, which is beneficial to the generation and diffusion of photogenerated carriers. Therefore, they are suitable for use as photocatalytic materials and short-wavelength optoelectronic devices. One-dimensional ZnO nanomaterials are characterized by high aspect ratio, high mechanical strength and high thermal conductivity. The carrier migration is along the *c*-axial direction and the carrier transport speed is fast, which can effectively reduce the electron-hole composite probability and greatly improve the photoelectric conversion efficiency. In addition, the high aspect ratio makes the piezoelectric effect of one-dimensional ZnO more obvious and it is suitable for pressure sensors. Two-dimensional ZnO nanomaterials are generally oriented preferentially along the *c*-axis and they have high acoustic–electric conversion efficiency, which is suitable for high frequency surface acoustic wave (SAW) devices. Three-dimensional ZnO nanomaterials usually have stereostructure and high specific surface area, which makes ZnO possess more hanging bonds and oxygen defects. It is helpful to improve surface activity and make it more sensitive to light and atmosphere. As a result, they are very suitable for the preparation of gas sensors and photodetectors.

**Figure 2:**
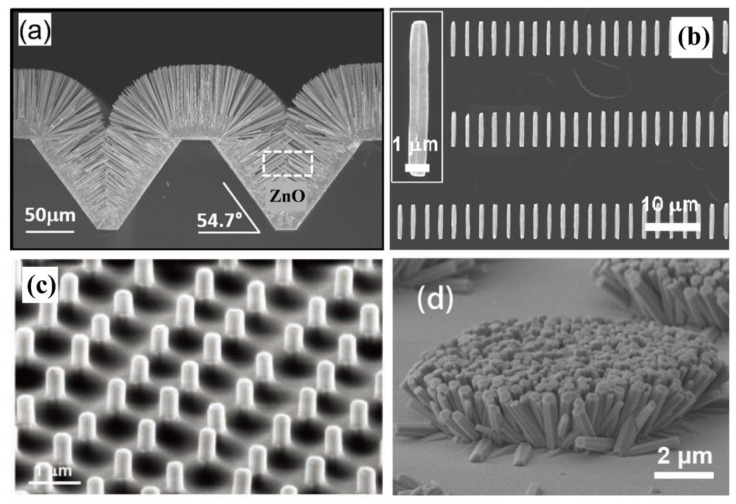
SEM images of ZnO nanostructure arrays. (**a**) ZnO nanocluster. Reproduced with permission [23]. Copyright 2013, American Chemical Society. (**b**) ZnO NR horizontal array. Reproduced with permission [24]. Copyright 2009, American Chemical Society. (**c**) ZnO NW vertical array. Reproduced with permission [25]. Copyright 2010, WILEY. (**d**) ZnO nanocluster arrays. Reproduced with permission [26]. Copyright 2015, WILEY.

With the development of miniaturization and high integration of electronic devices, it is the future trend to integrate a large number of sensors on chip, which makes the demand for nanosensor arrays increase drastically. Therefore, regular arrays of ZnO nanostructure have been fabricated through various synthesis methods. Figure 2 illustrates that, single ZnO nanowire in horizontal or vertical direction and nanoclusters can all be prepared into regular arrays, which provides a solid material basis for research on ZnO sensor arrays. 

ZnO nanomaterial used in sensors usually requires large specific surface areas, which contributes to increasing the contact area between the substance to be detected and semiconductor materials, thereby enhancing the performance of the sensors [27]. As a result, optimizing the ZnO nanomaterial synthesis method to further increase the specific surface area has become a hot spot in the past several years.

### 2.2. ZnO Nanomaterial Synthetic Method

There are various methods for synthesizing ZnO nanomaterials; the conventional means include the hydrothermal method [28], electrochemical deposition [29], magnetron sputtering deposition [30] and chemical vapor deposition [31], as well as sol-gel process [32]. Table 1 shows the comparison of some synthetic methods of ZnO nanomaterials.

Hydrothermal synthesis is a good approach for the synthesis of one-dimensional and two-dimensional ZnO nanostructures, with or without ZnO seeds on the substrate, which uses a precursor solution containing zinc ions and synthesizes at 60–100 °C for about 0.5–10 h. The size and morphology of products can be controlled by adjusting the concentration of the solution, reaction temperature and time. The hydrothermal method has many advantages, such as environmental friendliness, simple equipment and easy operation. In addition, this method is suitable for almost all substrates, because the growth environment is mild and the growth temperature is relatively low. However, the hydrothermal method also has many shortcomings [33]. For example, crystal defects in the synthetic material are unavoidable, due to the low growth temperature.

The electrochemical deposition method uses an electrolyte solution containing zinc ions in a closed loop. By applying voltage to the anode and cathode, the electron transfer and chemical reaction occur in the electrolyte solution, so that the target ZnO nanomaterials can be obtained on the substrate. This method is often used to synthesize ZnO thin film and the thickness, morphologies and the properties of deposited ZnO film can be controlled by adjusting the electrochemical parameters, such as the applied voltage signal, deposition time and ionic concentration [34]. However, electrochemical deposition is mostly used for the deposition of soluble substances. Due to the complexity of electrolyte and the existence of other chemical reactions in the growth of ZnO materials, the chemical composition of the synthetic ZnO nanomaterials is not uniform, which significantly affects the crystal quality of the prepared ZnO.

The magnetron sputtering method is often used to fabricate ZnO film, which uses the oxygen plasma to bombard the Zn or ZnO target material, then deposits ZnO on the substrate. This method has the superiorities of fast deposition speed, low temperature rise of substrate, high compactness and bond strength. The morphology, thickness and properties of the prepared ZnO film can be controlled by regulating cavity pressure, deposition time and temperature of substrate. However, ZnO fabricated by magnetron sputtering also has a series of problems, such as large surface roughness, poor uniformity and insufficient compactness [35].

Chemical vapor deposition is a common method for preparing one-dimensional ZnO nanomaterials. It consists of heating powder of a reactant containing Zn at high temperature to generate Zn vapor and, after a series of chemical reactions, to produce ZnO, which is deposited on the substrate. Depending on the used raw reactant materials, the reaction temperature ranges from 600 to 1200 °C. The morphology and size of the ZnO nanostructure are affected by the synthesis temperature, pressure and the position of the substrate in the quartz tube. ZnO synthesized by chemical vapor deposition at high temperature tends to possess high crystal quality [36], but the high temperature process may cause damage to the substrate, which makes it unsuitable for some flexible polymer substrates [37].

The sol-gel method is suitable for the synthesis of zero-dimensional ZnO nanomaterials, in which the Zn-containing compounds are dissolved by a solution and the sol system is formed after chemical reaction, then transformed into a gel system. Finally, after dehydration and heat treatment, ZnO nanoparticals are produced, which possess high purity and uniform distribution and the particle size of which is controllable [32]. However, the complex reactions, various variables and long process time are the disadvantages of this method, which seriously affect the performance of ZnO products.

An innovative way to prepare ZnO nanomaterial is by means of metal-organic frameworks (MOF), which are a new type of material with large specific surface area, adjustable pore size and easy functionalization of organic ligands and skeleton metal ions. This method usually uses organic reagents and Zn^2+^ ion to generate an MOF-structured ZnO precursor; then, it is calcined at high temperature to remove organic components, thus forming the porous ZnO nanostructures [38,39]. Therefore, ZnO fabricated by this method usually possesses a large surface area, which is very suitable for the fabrication of and research on sensors. Nevertheless, the purity of ZnO may be seriously affected by the presence of residual organic matter.

In summary, ZnO nanomaterials with various morphology characteristics have been synthesized through different methods [40,41]. Moreover, the preparation technologies are relatively mature, after the systematic research, which provides a sound material foundation for the intensive study of ZnO nanosensors [42].

## 3. Research Progress of ZnO Nanosensor

ZnO nanostructure has excellent piezoelectric, optical and electrochemical properties. Based on different working principles, various ZnO nanosensors have been developed, such as pressure sensors [43], gas sensors [39], photoelectric sensors [44] and temperature sensors [45], as well as biosensors [46]. In the following sections, we will overview the recent development of ZnO nanosensors according to the functions and application fields [47,48].

### 3.1. ZnO Pressure Sensors

#### 3.1.1. Mechanism of ZnO Pressure Sensors

The piezoresistive and piezoelectric characteristics of ZnO make it particularly sensitive to pressure signals; hence, plenty of pressure sensors based on ZnO nanostructures have been investigated recently [49,50].

When strain is applied, the energy band of ZnO changes, resulting in the variation of resistivity, which is the piezoresistive effect of ZnO. A ZnO piezoresistive pressure sensor converts the external mechanical stimuli into electrical signals based on the piezoresistive effect and this is the mechanism of a ZnO piezoresistive pressure sensor [51].

ZnO creates a piezoelectric potential inside the crystal under external strain, due to the non-central symmetric wurtzite structure. The inner-crystal piezopotential could tune the charge generation, separation, transportation and recombination, thus affecting the electrical properties of ZnO devices. ZnO possesses a strong piezoelectric coefficient (12.4 pC N^−1^), compared to other semiconductors [52], especially for ZnO with high quality and C-axis preferred orientation, which has been widely used in pressure signal sensing. This is the mechanism of a ZnO piezoelectric pressure sensor.

#### 3.1.2. Progress in ZnO Pressure Sensors

Pressure sensors based on ZnO nanomaterials have been investigated for a long time and the ZnO nanostructures used in pressure sensors have been gradually optimized for different application scenarios.

The earliest pressure sensor was made of ZnO film, since ZnO film can be prepared easily and it has high stability [53]. The Z. L. Wang group proposed a flexible piezoelectric pressure sensor based on ZnO film [54]; and they elaborated the piezotronics effect over the geometry of the as-fabricated devices and the modulation effect of piezopotential on charge carrier transport under different strains. However, it is difficult to achieve high sensitivity for pressure sensors based on ZnO film, because the piezoelectric characteristic is not obvious due to the inconsistent orientation of the crystals inside the film structure.

**Figure 3:**
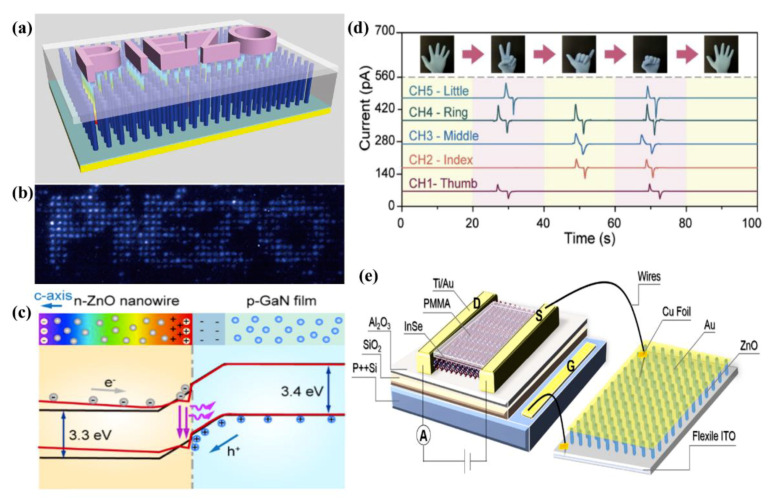
Pressure sensors based on ZnO nanostructure. (**a**,**b**) Pressure sensor based on a ZnO NW array on rigid GaN substrate. Reproduced with permission [58]. Copyright 2013, Nature Publishing Group. (**c**) Pressure sensor based on a ZnO NW array on flexible GaN substrate. Reproduced with permission [59]. Copyright 2019, Elsevier. (**d**) Pressure sensor based on Sb-doped p-ZnO NW films. Reproduced with permission [56]. (**e**) FET Pressure sensor based on ZnO NR arrays. Reproduced with permission [60]. (**d**,**e**) Copyright 2020, Elsevier.

In contrast, one-dimensional ZnO exhibits superior piezoelectric properties and it is more suitable for pressure sensor. Single ZnO NW pressure sensors were first studied in 2013, when the Z. L. Wang group fabricated a pressure sensor based on an ultralong Sb doped p-type single ZnO NW, which was up to 60 μm in length. They further confirmed the existence of the piezotronic effect in p-type ZnO NWs films and realized strain-gated piezotronic transistors [55]. Recently, the C. F. Pan group took advantage of the Sb-doped p-ZnO NW films to achieve self-powered piezoelectric pressure sensors, which not only can detect the motion of each finger individually and accurately, but can also evaluate change in gestures based on the output signal [56], as shown in Figure 3d. Single ZnO NW and ZnO NW films are both proved to be high efficiency sensor materials. In addition, ZnO NRs are usually used in pressure sensors. Y.S. Tan et al. proposed a textile piezoelectric pressure sensor based on ZnO NR arrays for wearable application, which exhibited excellent performance, including a low detection limit of 8.71 Pa, high output voltage of 11.47 V and superior mechanical stability. The sensitivity of the sensor was 0.62 V·kPa^−1^ in the pressure range of 0–2.25 kPa. Their work demonstrated that ZnO-based wearable sensors have great application potential in the future [57].

With the development of integrated circuits and wearable electronics, pressure sensor arrays with high integration have aroused wide concern [61]. ZnO NW arrays are the preferred materials for preparing pressure sensor arrays due to their high density, high uniformity and easy adjustment. C. F. Pan et al. reported a high resolution pressure sensor based on a ZnO NW LED array, which can map two-dimensional distributions of strain and achieve an unprecedented spatial resolution of 2.7 μm, as shown in Figure 3a,b [58]. In 2019, this group further explored flexible pressure sensors based on ZnO nanowire LEDs, realizing a high spatial resolution of 2.6 µm and a fast response time of 180 ms [59], as shown in Figure 3c. It still worked well after 4000 bending circles, which demonstrates that the flexible pressure sensor exhibited excellent mechanical stability. These characteristics are excellent in the field of flexible pressure sensor arrays, in addition to the advantage of the visual. Both of the above studies took advantage of the piezoelectric charges in n-type ZnO nanowires induced by applied strain, which could significantly affect the charge carrier separation and transport at the interface/junction of ZnO/GaN, so as to regulate the LED luminous intensity and realize the visual pressure sensor array. Their studies provided new ideas for the application of visual stress sensors with either rigid or flexible substrates.

In addition, the application of ZnO nanomaterials in field-effect transistors (FET) is becoming more and more mature [62]. Recently, F. L. Wang et al. fabricated flexible two-dimensional FET with ZnO NR arrays and a 2D indium selenide (InSe). The highlight of this study is that they took the piezoelectric voltage of ZnO NR as gate potential, which can be effectively amplified by the FET, then affected the current in FET, thus realizing a pressure sensor with a sensitivity of 19.6 Pa, corresponding to 0.1 g of loading [60], which was superior to that of other researches, as described in Figure 3e. Their work showed that the piezoelectric properties of ZnO nanostructures can be utilized in both pn structure and FET structure devices to realize pressure sensors with high performance.

#### 3.1.3. Discussions

In conclusion, pressure sensors based on ZnO nanomaterials have experienced rapid development from thin film and single nanowire to nanowire/nanorod arrays [63]. Due to the larger aspect ratio, one-dimensional ZnO nanomaterials possess a more remarkable piezoelectric effect. Therefore, one-dimensional ZnO nanomaterials, such as ZnO NW, NR and NB, are more widely used in the field of pressure sensors. Pressure sensors based on ZnO NW arrays with ultra-high resolution and sensitivity have been studied maturely, which provides innovative ideas for modern applications, such as tactile sensing, human–computer interaction and measurements of stress in the micro/nano region [64]. However, there are still some problems in pressure sensors based on ZnO nanomaterials, such as how to optimize the synthesis method to fabricate nanostructure arrays with fewer crystal defects and higher uniformity at low temperature, which affects the performance of pressure sensors and remains a challenging technological problem.

### 3.2. ZnO Gas Sensors

#### 3.2.1. Mechanism of ZnO Gas Sensors

Gas sensors play an important role in gas detection, automatic control, danger alarm and other application fields, which can convert gas information into electrical signals. According to the working mechanism, gas sensors can be divided into resistive sensors and non-resistive sensors and most ZnO gas sensors belong to the former. ZnO, as an n-type semiconductor, is a key member for gas sensors, due to its superiority of low cost, low toxicity, good thermal stability and high electron mobility [65]. When the gas to be measured is adsorbed on the surface of the ZnO material, the resistivity of ZnO changes accordingly, leading to the gas-sensitive property. Especially with ZnO nanomaterials, which possess high surface-to-volume ratio, they could absorb more gas molecules on the surface, resulting in a remarkable change of surface resistance, thus making the gas sensor more sensitive. As a result, ZnO nanomaterials contribute to improving the performance of gas sensors significantly. ZnO materials are sensitive to carbon monoxide, ethanol, hydrogen, nitrogen dioxide and other gases [66,67]. When ZnO adsorbs a reductive gas, the resistivity decreases with the increase of gas concentration. On the contrary, when oxidizing gas is adsorbed, the resistivity increases with the increase of gas concentration. ZnO gas sensors work efficiently based on this theoretical mechanism.

#### 3.2.2. Progress in ZnO Gas Sensors

Various ZnO gas sensors have been researched extensively and the detected gases include CO, NO_2_, NH_3_, H_2_S, ethanol, acetone, formaldehyde and so on [68,69]; according to the characteristics of the used ZnO materials, they can be roughly divided into conventional n-type ZnO nanomaterials, specially doped ZnO nanomaterials and some ZnO complexes. In addition, some studies have shown that suitable irradiation could enhance the performance of ZnO gas sensors.

**Figure 4:**
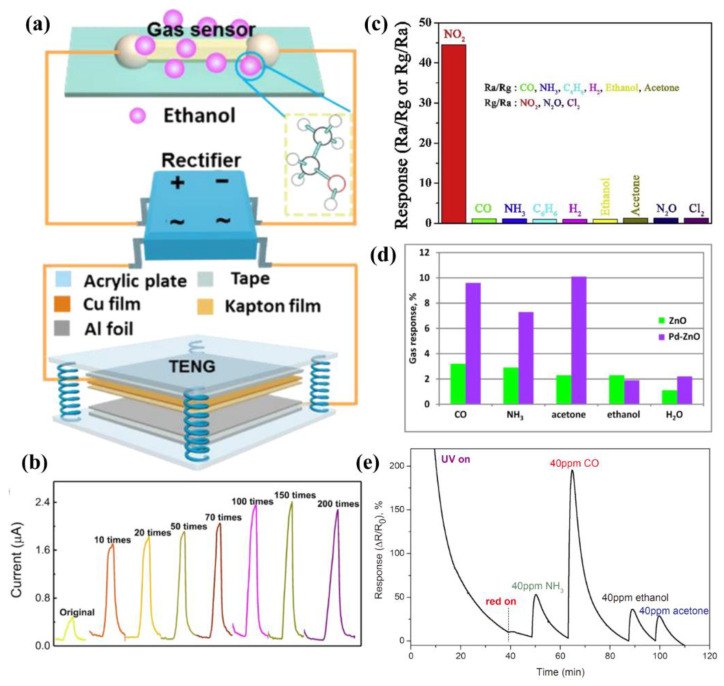
Gas sensors based on ZnO nanostructure. (**a**,**b**) ZnO NMW gas sensor for ethanol. Reproduced with permission [72]. (**c**) ZnO/g-C_3_N_4_ composite gas sensor for NO_2_. Reproduced with permission [80]. (**d**) Pd-ZnO gas sensor. Reproduced with permission [78]. (**e**) Irradiation-assisted gas sensor. Reproduced with permission [79]. Copyright 2020, Elsevier.

First, gas sensors based on conventional n-type ZnO have always been widely reported [70,71]. J. P. Meng et al. proposed a highly sensitive ethanol sensor based on Schottky junction, prepared with a single n-type ZnO nano/microwire (NMW); the response of the sensor was 4.8 and it was enhanced by 139% for 100 ppm ethanol after introducing a triboelectric nanogenerator to lower the Schottky barrier height [72], as shown in Figure 4a,b. This research proves that a single ZnO nanowire could be manufactured as a micro/nano gas sensor with high response, which would greatly accelerate the miniaturization development of sensors. Moreover, their work introduces an effective method to improve the responsiveness of gas sensors by using a triboelectric nanogenerator, which provides an innovative idea for the development of other types of sensors.

In order to improve the performance of gas sensors, some specially doped ZnO nanomaterials have been proved to exhibit excellent gas sensing properties [73]. A number of studies have shown that Cu- or Pt-doped ZnO nanostructures are sensitive to H_2_ [74,75] and Au-, Ag- or Pd-doped ZnO nanostructures are sensitive to ethanol and acetone [76,77]. Recently, T. Dilova et al. compared the gas sensors fabricated with pure and a palladium (Pd)-doped ZnO nanobelt [78]. As shown in Figure 4d, the results showed that the Pd-ZnO nanosensor showed a better response to CO, NH_3_ and acetone than the pure ZnO sample, which mainly owes to the Pd nanoparticles decorated on the ZnO surface increased number of adsorption centers. In the same year, they prepared nanosensors with highly porous pure and silver (Ag)-doped ZnO nanostructures and studied their response to NH_3_, CO, ethanol and acetone under the irradiation of ultraviolet light and infrared light. It was found that when the infrared light was irradiated, the response of the sensor to CO was obviously enhanced, but the response to acetone and ethanol was weak, as shown in Figure 4e [79]. Under the irradiation of an ultraviolet light intensity of 43 MW cm^−2^, the sensor had the highest response to acetone and ethanol and the lowest response to CO. They also studied the influence of ultraviolet and red irradiation on the response and response recovery time of sensor components. The results showed that ultraviolet and optimized red irradiation enhanced the response of the Ag-ZnO sensor to CO, while suppressing its response to NH_3_, ethanol and acetone. Moreover, the sensor showed a strong and stable response signal under a CO concentration of 1 ppm, which indicates that a low concentration of CO can be detected by simultaneous irradiation of ultraviolet light and infrared light. Their work mainly verified the influence of light irradiation on the performance of gas sensors, which lay the research foundation for the development of high performance and multi-function sensors.

In addition, the research on the ZnO complex for gas sensing is increasingly extensive. H. T. Wang et al. fabricated a sensor with a ZnO/g-C_3_N_4_ composite and studied its sensing performance for NO_2_ under different wavelength LED light sources, as shown in Figure 4c [80]. Under the illumination of 460 nm wavelength, the maximum response of ZnO/g-C_3_N_4_ to 7 ppm NO_2_ was 44.8 and the detection limit was 38 μg L^−1^, with a response time of 142 s and recovery time of 190 s. The highlight of this research is that sensors with ZnO/g-C_3_N_4_ composites exhibited better selectivity and high stability, which is attributed to the absorbance of g-C_3_N_4_ in the visible light region and the charge separation at the interface between ZnO and g-C_3_N_4_. F. F. Cao et al. compounded carbon nanotubes (CNT) on ZnO nanorods (ZnONR) by ultrasonic dispersion and studied two kinds of gas sensors prepared with ZnONR and ZnONR/CNT, respectively [81]. They found that these two kinds of gas sensor exhibited good selectivity, stability and reproducibility for 0.01% mass fraction of ethanol at 370 °C and, in contrast, sensors with ZnONR/CNT showed superior performance due to the hollow tubular structure, which greatly increases the surface area of nanomaterials. These studies declared that by combining ZnO with other nanomaterials with a high specific surface area, the gas absorbance can be further increased, which is an effective way to improve the sensitivity and response of ZnO gas sensors.

#### 3.2.3. Discussions

In brief, gas sensors based on ZnO nanomaterials show excellent properties and, through combining with other nanomaterials with high specific surface areas or embellished with metallic elements, ZnO exhibits increased surface area, which helps to improve the response of the gas sensor [82]. High selectivity, which means that they can accurately identify the target gas to be measured in complex mixed atmosphere, is an important parameter for gas sensors. The selectivity of ZnO gas sensors can be improved effectively by modifying the surface of ZnO materials with functional groups or metals. In addition, the proper irradiation could also affect the sensitivity of ZnO gas sensors, due to the change in the vacancy stage introduced by irradiation. ZnO gas sensors have attracted much attention due to simple preparation, stable properties and good compatibility [83]. However, gas sensors based on ZnO nanostructures still face many obstacles, such as high working temperature, high resistivity, poor selectivity and low sensitivity. Hence, there is still a lot of work to be conducted for the realization of gas nanosensors with superb performance in the future.

### 3.3. ZnO Photoelectric Sensors

#### 3.3.1. Mechanism of ZnO Photoelectric Sensors

Photoelectric sensors can convert optical signals into electrical signals and have been widely used in industrial automation and robots due to the advantages of non-contact, fast response and high precision. Various photoelectric sensors have been proposed, mainly including infrared (IR) sensors, ultraviolet (UV) sensors and visible wavelengths sensors, according to the working wavelength [84].

Due to the wide bandgap, low cost and simplicity in manufacturing process, ZnO has become a potential semiconductor for photoelectric sensors and it has aroused extensive investigations [85,86]. When under illumination, as long as the photon energy is larger than the bandgap width of ZnO, the electrons in the valence band could absorb the energy of the photon, then transition to the conduction band, generating additional free electrons and holes. The resistivity of ZnO semiconductors becomes smaller due to the photogenerated carriers, which affects the electrical signals in the photoelectric device. This is the mechanism of ZnO photoelectric sensors. According to their typical wide bandgap (3.37 eV) and large exciton binding energy (60 meV), ZnO nanostructures are suitable for detecting UV light [87]. In addition, ZnO nanomaterials are usually used to prepare infrared photoelectric sensors because of their high electron mobility and regular nanostructures.

#### 3.3.2. Progress in ZnO Photoelectric Sensors

##### UV Photoelectric Sensor

In recent years, UV photoelectric sensors based on ZnO nanomaterials have received more and more attention, including ZnO films, single ZnO NW, ZnO NW arrays and ZnO complexes. Then, we display a brief introduction to the UV photoelectric sensors according to the morphology of ZnO nanomaterials.

**Figure 5:**
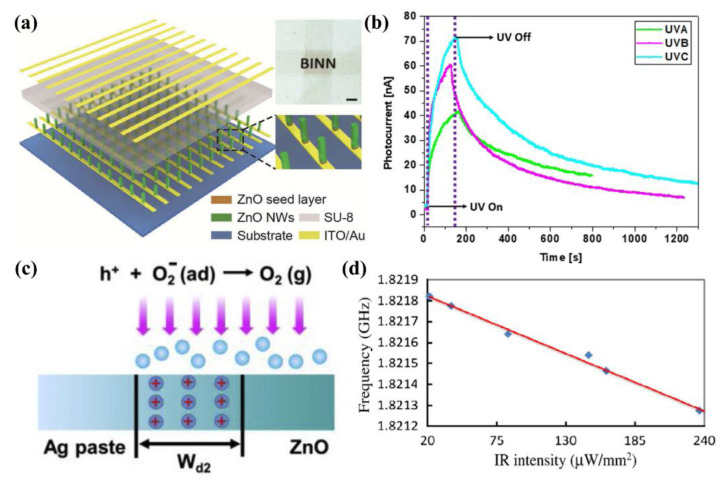
Photoelectric sensors based on ZnO nanostructures. (**a**) ZnO NW UV sensor arrays. Reproduced with permission [89]. Copyright 2015, WILEY. (**b**) ZnO bi-layer structure UV sensors. Reproduced with permission [88]. Copyright 2020, Elsevier. (**c**) ZnO UV sensor based on Schottky junction. Reproduced with permission [90]. Copyright 2020, Elsevier. (**d**) IR sensor based on ZnO film. Reproduced with permission [91]. Copyright 2011, Elsevier.

UV photoelectric sensors based on ZnO film have been investigated widely. Recently, M. Cavas et al. investigated two types of photoelectric sensors based on pure ZnO film and Ga-doped ZnO film on a p-Si substrate, respectively. The results of their research show that the bandgap of ZnO varies with Ga doping concentration, which has a great influence on the photoelectric performance of the device. They proved that the optimal Ga-doping ratio is 5% and the UV photoelectric sensor represents the best photoelectric sensing characteristics, compared with that of devices in other doping ratios [87]. M. B. Jeon et al. demonstrated a port-resonator-types of UV sensor consisting of Ag NW and ZnO thin film bi-layer, with a resonance frequency of 243.15 MHz. Their research proved that the UV irradiation-generated photocurrent influences the acoustic-electric effect of the SAW sensor, as shown in Figure 5b [88]. Compared to sensors with a ZnO single thin film, bi-layer structure sensors exhibited a more excellent response and recovery characteristics, due to the electron trap center within the ZnO film compensated by the AgNW/ZnO structure and the larger surface area for oxygen adsorption.

ZnO NW is a good material for preparing photoelectric sensors and a number of studies have been reported in recent years [92,93]. H. Li et al. reported a UV sensor based on the Schottky barrier height (SBH) of single ZnO NW and Ag paste, as illustrated in Figure 5c [90]. Moreover, they further enhanced the response characteristics through lowering the SBH using triboelectric voltage, thus finally achieving a raising edge of 1.05 s, a falling edge of 0.38 s and an on/off ratio of up to 10,400. This work presented a simple structure of UV sensors based on single ZnO NW with excellent response and proposed an innovative method to improve its performance. In addition, UV sensors based on ZnO NW arrays have been deeply studied. Recently, C. F. Pan et al. proposed a ZnO NW UV sensor arrays consisting of 32 × 40 pixels, with spatial resolution of 100 µm (254 dpi); when under 3.95 mW cm^−2^ UV illumination, the measured response time was 62 ms, as shown in Figure 5a [88]. Then, they improved the performance of the UV sensor by introducing the piezo-phototronic effect, including a 700% increase in photoresponsivity and 600% increase in sensitivity. In this study, UV sensor arrays with high responsiveness were successfully realized by using both photoelectric and piezoelectric characteristics of ZnO NWs.

ZnO complexes have been used in the field of photoelectric sensors and present splendid properties. Y. F. Zhao et al. fabricated three types of UV sensors using pure NaTaO_3_, ZnO and their composites, respectively [94]. Among them, a UV sensor based on 1:1 NaTaO_3_/ZnO displayed the most outstanding photoelectric performance, including a low dark current, a high photo-to-dark current ratio and a stable periodic photoresponse. Their research showed that a ZnO compound with appropriate proportions has a potential application value in improving the performance of photoelectric sensors.

##### IR Sensor

In addition to the UV photoelectric sensor, IR sensor based on ZnO nanomaterials have aroused wide concern recently [95,96]. ZnO nanostructures mainly act as an electron transport layer or an optical resonator with high quality in these photoelectric sensors.

Recently, J. B. Kwon et al. provided a shortwave infrared (SWIR) sensor based on lead chloride quantum dots (PbS QDs) and ZnO NPs, in which PbS QDs worked as the photoactive layer and ZnO NPs worked as the electron-transport layer [85]. It was found, by comparing PbS SWIR sensors with and without a ZnO NPs layer, that the former exhibited higher on/off ratio and more stable current characteristics, due to the high electron mobility and the lowest unoccupied molecular orbital level of ZnO NPs.

In addition, Z. Y. Wang et al. fabricated a film bulk acoustic resonator (FBAR) based on ZnO thin film. Figure 5d shows that the resonant frequency of FBAR was linearly related to infrared light intensity under the irradiation of infrared light and the resonant frequency decreased with the increase in infrared light intensity. This is because the Young’s modulus of the ZnO resonator is affected by temperature, thus leading to the shift in resonant frequency. Their study confirmed the feasibility of FBAR as a potential infrared detector, which shows an excellent response to infrared light [91].

#### 3.3.3. Discussions

A large number of experiments has proved that ZnO nanostructure is one of the ideal materials for preparing photoelectric sensors. No matter whether it is pure ZnO nanostructure, doped ZnO nanomaterials, or ZnO composite materials, they all exhibit favorable photoelectric properties. Although the energy band of ZnO only corresponds to the UV light wavelength, ZnO can also play an important role as an electron transfer layer in the photoelectric sensor for other light wavelength ranges. For example, the Z. L. Wang group demonstrated a flexible visible/UV photodetector, which consisted of a ZnO-CdS double-shell NW array and carbon fiber. It presented ultrahigh response to blue, green and UV light; in addition, the responsivity was enhanced by 60% under a −0.38% compressive strain due to the piezopotential in ZnO [97]. Their study verified that ZnO, as preferred material for photoelectric sensor, not only has a wide sensitive band, but also possesses piezoelectric characteristics, which could help improve the photoelectric sensing performance.

However, photoelectric sensors based on ZnO still face some pressing problems, such as the high resistivity of ZnO, which leads to a low photocurrent and poor detection performance of the photoelectric sensor [98]. Moreover, the question of how to break through the limitation that the band gap of ZnO just corresponds to the ultraviolet band, which is a tough problem, needs to be solved. In addition, the sensitivity to light largely depends on the shape and size of the ZnO nanostructure [99], but the research on different morphologies and structures of ZnO is still immature, which is also a major factor restricting the application of ZnO-based photoelectric sensors.

### 3.4. ZnO Biosensors

#### 3.4.1. Mechanism of ZnO Biosensors

In recent years, biosensors have attracted extensive attention from all walks of life and have been widely used in the detection of protein, hormones and peptides [100]. The mechanism of biosensors is that when the molecules to be detected enter the bioactive material through diffusion, a biological reaction occurs and the generated information is converted into electrical signals to instruct the concentration of the substance to be measured [101]. ZnO nanomaterials are commonly used in biosensors, since they possess outstanding biocompatibility, electron transfer characteristics, wide bandgap, large surface area and mature synthesis techniques [102]. In general, ZnO plays the role of increasing the adsorption area of biomolecules and promoting electron transfer in biosensors, which can effectively improve the responsivity and sensitivity of biosensors.

#### 3.4.2. Progress in ZnO Biosensor

Biosensors based on ZnO nanomaterials have been widely studied recently [103] and, next, we provide a brief introduction to biosensors according to the morphology of ZnO in the biosensor.

Zero-dimensional ZnO nanomaterials have been proved to be a suitable material for biosensors. U. D. Kamaci et al. demonstrated a ZnO QDs-based fluorescent biosensor for detection of cysteine in different solutions, which exhibited a high selectivity and responsivity [104]. Another research used ZnO/NiO/Al_2_O_3_ nanoparticles to realize an enzyme-less l-glutamic acid (L-GA) sensor with superior sensing performance [100]. These studies show that zero-dimensional ZnO nanomaterials play an important role in biosensors due to their large surface area.

**Figure 6:**
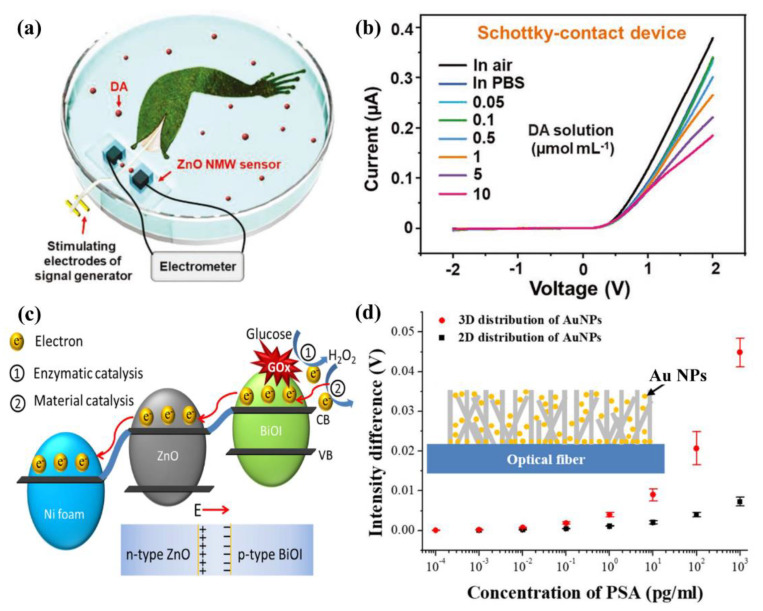
Biosensors based on ZnO nanostructure. (**a**,**b**) ZnO NMW biosensor for dopamine. Reproduced with permission [105]. Copyright 2019, WILEY. (**c**) ZnO/BiOI core-shell biosensor for glucose. Reproduced with permission [106]. Copyright 2020, Elsevier. (**d**) ZnO NWs biosensor for PSA. Reproduced with permission [107].

Single ZnO NW has been investigated to form efficient biosensors. L. M. Zhao et al. proposed a highly sensitive multifunctional biosensor based on Schottky and Ohmic reversible (SOR) structures, which can convert between Schottky and Ohmic contact tuned by triboelectric nanogenerator (TENG) due to the diffusion of oxygen vacancies in ZnO nano/micro wire (NMW) when it is driven by TENG voltage pulses [105], as shown in Figure 6a,b. In the Schottky contact state, the biosensor is sensitive to low concentration of dopamine (0.1 μmol mL^−1^), while, in the ohmic contact, it exhibits sensitivity to neural electric impulses. Therefore, this work demonstrated a comprehensive multifunctional sensor, which will broaden the application of biosensors for brain science and clinical diagnosis.

ZnO NW arrays have been proved to be a suitable material in biosensors. H. M. Kim et al. fabricated a highly sensitive plasmonic biosensor based on three-dimensional (3D) structures composed of optical fiber, ZnO NWs and Au NPs [107]. Compared with biosensor with 2D structures, the sensitivity to prostate-specific antigen (PSA) of 3D biosensors was improved notably, as illustrated in Figure 6d. This is because the introduced ZnO NWs helped focusing light on the sensing region due to its forest-like structural characteristics and enhancing the surface area for attaching Au NPs. Furthermore, the porous structure of ZnO NWs constitutes a fast channel for target molecules accessing the surface of the Au NPs.

In addition, the ZnO complex is one of the most suitable materials for biosensors. M. G. Zhao et al. reported a glucose biosensor composed of Ni foam and the ZnO/BiOI core-shell p-n junction nanorods, which played an important role in working as an enzyme loading matrix for detecting glucose, as described in Figure 6c [106]. The composite structures contributed to enhancing the performance of the biosensor, with an enhanced sensitivity of 115.2 μA·mM^−1^·cm^−2^ and a decreased detection potential of 0.3 V, because it provided multiple catalysis and the porous surface helped loading more enzymes. Another significant advantage of this design structure is that the p-n junction accelerated electron transport due to the existence of the built-in electric field and the electron well. Another research carried out by S. Pal et al. demonstrated a DNA hybridization biosensor based on surface plasmon resonance (SPR), consisting of a SF10 prism-Au-ZnO-Graphene-PBS solution [108]. Since both ZnO and graphene possess excellent chemical stability, light absorption capability and biocompatibility, the DNA hybridization biosensor presented outstanding sensitivity.

#### 3.4.3. Discussions

Numerous studies have demonstrated that the combination of ZnO nanostructures and biosensors not only broadens the application field of semiconductor nanomaterials, but also promotes the development of biosensor technology in various fields [109,110]. For example, the composite of Ag and ZnO nanomaterial exhibited good antibacterial property and the composite materials of ZnO doped with graphene were applied to the detection of special DNA. Although ZnO could greatly promote electron transfer in biosensors, there are still some bottlenecks for the ZnO biosensors. One of the severe problems is that the surface control of ZnO nanomaterial and the fusion with other doping materials have a great influence on the sensitivity of the sensor, which significantly limits the development of ZnO-based biosensors. Therefore, on the one hand, enhancing the characteristics of ZnO, such as further increasing its specific surface area and electron transfer rate, and, on the other hand, improving the integration between ZnO and other materials, especially bio-organic materials, are the scientific problems to be explored for ZnO biosensors in the future. Improving the stability, repeatability, selectivity and sensitivity of the biosensor by the surface modification method has been the main research of ZnO biosensors in the past few decades and it remains to be the focus of future research. It is a significant challenge to explore and discover the sensitivity of different ZnO structures to various biological systems and to realize biosensors with more functions, so as to provide a theoretical basis for the development of health care and detection field.

### 3.5. ZnO Temperature Sensors

#### 3.5.1. Mechanism of ZnO Temperature Sensors

A temperature sensor converts the temperature signal into an electrical signal and its working principle is that the resistance value and the potential of the thermocouple change regularly with the variation of temperature. Thus, temperature information can be displayed by monitoring the electrical signals, since the carrier concentration and the energy gap of ZnO changes with temperature, resulting in regular variation of electrical properties accordingly. Therefore, ZnO is an excellent material suitable for preparing temperature sensors, which have been investigated for a long time [111].

#### 3.5.2. Progress in ZnO Temperature Sensor

In recent years, temperature sensors based on ZnO have been proposed successively [112], including sensors based on ZnO nanostructures, ZnO-decorated optical fiber and SAW devices. Here, we give a brief overview of these ZnO temperature sensors, respectively.

##### ZnO Nanostructures Temperature Sensor

**Figure 7:**
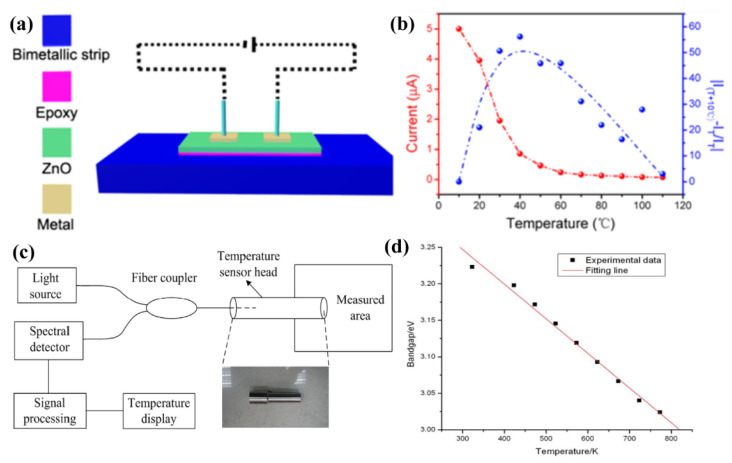
Temperature sensors based on ZnO nanostructure. (**a**,**b**) Flexible temperature sensor based on ZnO NW film. Reproduced with permission [113]. Copyright 2014, American Chemical Society. (**c**,**d**) Fiber optical temperature sensor based on ZnO NW film. Reproduced with permission [114]. Copyright 2014, Elsevier.

ZnO nanomaterials with various morphologies have been widely used in the preparation of temperature sensors. F. Xue et al. demonstrated a flexible temperature sensor based on ZnO NW film, which was fabricated by wet chemical deposition, as shown in Figure 7a [113]. Piezopotential in ZnO NW film contributed to enhancing the sensitivity of the sensor and the red line in Figure 7b indicates that the response current of the sensor decreased as the temperature increased from 10 to 110 °C. This is because the piezoelectric effect increases the Schottky barrier, which reduces the current in the temperature sensor. The blue line shows the relative current change, which indicates a well response of this temperature sensor. Their work provided a simple temperature sensor model and an effective method to improve the performance of ZnO temperature sensors.

In addition, G. H. Hui et al. used ZnO nanoparticles and Au interdigital electrodes to realize a temperature sensor [115]. The results show that the current decreased with the temperature increase, due to the reduction of electrical carrying body, including oxygenic cavity and Zn spacing. Furthermore, the temperature sensor presented excellent sensitivity and repeatability. Their research proved that the defect states in ZnO could improve the sensing performance of the temperature sensor to some extent.

##### ZnO SAW Temperature Sensor

SAW temperature sensors based on ZnO are one of research hotspots [116]. A flexible SAW temperature sensor was reported by S. A. Hasan et al., composed of ZnO thin film and aluminium (Al) foil [117]. The device was measured on a printed circuit board (PCB), which can be flat or curved. The research proved that the flexible sensor steadily exhibited high sensitivity in both flat and curved conditions, which declares the excellent mechanical stability of the sensor and the application prospect in the wearable temperature sensing electronics. Another SAW temperature sensor made of ZnO and LiNbO_3_ substrate was reported by H. Yamamoto [118] and it showed that the temperature dependence on frequency exhibited linear characteristics. In conclusion, SAW temperature sensors based on ZnO possess good performance and they provide new ideas for the study of temperature sensors.

##### ZnO Optical Fiber Temperature Sensor

Fiber optic temperature sensors have wide application prospects in industrial production, aerospace and other fields, due to the advantages of low loss over long distances, flexibility, small size and light weight of optical fiber. Optical fiber temperature sensors with ZnO nanostructures have aroused research interest recently; they can measure the temperature by detecting the transmission spectrum and observing the movement of the absorption edge. They have the advantages of simple structure, convenient operation, low cost and wide temperature range.

Recently, H. L. Wang et al. proposed a fiber optical temperature sensor employing the spectral absorption effect of ZnO thin film, with a thickness of 500 nm [114]. The measured system can be seen in Figure 7c,d shows that the bandgap energy Eg of ZnO thin film decreases with the temperature increase. After optimizing the light source (47 nm) and annealing ZnO film in air at 673 K, the results show that the temperature sensor possessed a wide measurement range (373–873 K), a high resolution of 1 °C and a superior sensitivity of 0.06 m °C^−1^. Therefore, this temperature nanosensor is suitable to be applied in some more demanding environments. Another research, reported by C. H. Sui et al., presented an optical temperature sensor based on ZnO thin film which was only related to the wavelength shift of transmittance spectra. Further, it illustrated that a fiber optical sensor could work with a broad temperature measurement range of 10–1800 K, which shows the promising utilization potentiality of ZnO optical fiber temperature sensors [111].

#### 3.5.3. Discussions

As discussed above, ZnO temperature nanosensors have been studied a lot and the device structure and performance improved significantly. ZnO plays an important role in temperature nanosensors, because its carrier concentration and energy gap are sensitive to the temperature, which affects the conduction characteristics. Due to the faster electron transportation of one-dimensional ZnO nanostructures, they are more suitable for the preparation of temperature sensors. However, in order to meet the higher aggregate demand of future applications, there are still some key technologies for ZnO temperature sensors to be broken through. For example, the temperature measurement range and stability can be further optimized by regulating the quality of ZnO nanomaterials; the integration of the temperature sensor can be enhanced by compressing the device structure, which is a great challenge to device fabrication technology. Further, the heat capacity and maximum temperature that can be sustained by the device should be increased by exploring the temperature transfer and compensation between ZnO and the remaining structures of the device. Therefore, the future research on ZnO temperature sensor still remains abundant and arduous.

## 4. Conclusions and Prospects

In this review, firstly, ZnO nanostructures and the main synthetic methods of ZnO materials were introduced. Then, the development of several types of nanosensors based on ZnO were reviewed intensively, including pressure sensors, gas sensors, photoelectric sensors, biosensors and temperature sensors. The sensing mechanism, recent research progress and challenge problems were described for each type of sensor, respectively. All the above ZnO nanosensors show excellent sensitivity, responsivity and stability, due to the superior piezoelectric properties, photoelectric properties, electronic transmission and the large surface area of ZnO nanostructures. Therefore, the ZnO nanosensors have attracted wide attention not only in scientific research but also in various application fields.

Although favorable sensing performance has been achieved for some ZnO nanosensors in recent years, many challenges for commercial applications still remain to be overcome. First, the high-quality and stable p-type ZnO materials have become the bottleneck for material synthesis, which restricts the development of ZnO nanosensors seriously. In addition, it is a key difficult problem to precisely control the morphology of ZnO nanostructures, which plays a vital role in nanosensors. Moreover, how to further improve the selectivity and response of ZnO nanosensors is still a research direction worth exploring. Finally, there are many obstacles in realizing ZnO nanosensors with high integration and multi-function, which is the inevitable trend of development for electronic devices in the future. At present, the majority of ZnO sensors are single-function and only a few works have realized multi-function sensors at the same time [115]. For example, Y. Wang et al. demonstrated a pressure and light difunctional sensor based on ZnO NW/Graphene [116]. However, the realization of multifunctional sensors not only needs to solve the problems of integration process, but also needs to overcome the technical challenges of signal recognition, transmission and mutual disturbance when multiple physical quantities are measured simultaneously. Therefore, the research of multifunctional ZnO sensors is an enormous challenge.

In view of the above problems, the future research on ZnO nanosensors may focus on the following aspects. The synthesis methods of P-type ZnO and ZnO complex need to be further improved to provide comprehensive ZnO nanomaterials with high quality, which is an important foundation for the development of ZnO nanosensors. How to achieve the precise control of the surface of the synthetic nanomaterials is a key step to promote the preparation process of ZnO. The selectivity of ZnO sensors could be improved by introducing surface catalysts, modification with functional group and doping of ZnO, which would enhance the reception rate of the target detection objects. Meanwhile, the response of ZnO sensors could be improved by further optimizing the quality of ZnO nanomaterials and device structure. Under the premise of ensuring the performance, the main approaches to promote sensors moving towards high integration and multi-function are simplifying the device structure, reducing the physical size and increasing the compatibility of ZnO and other materials. Therefore, great and collective efforts are essential to address these challenges and we believe that ZnO nanosensors possess enormous potential for future applications in various electronic systems and artificial intelligence.

## Figures and Tables

**Table 1:** Synthetic methods of ZnO nanomaterial.

Synthetic Method	Synthetic Environment	Advantage	Disadvantage
Hydrothermal	solution environment;	environmental friendliness; simple equipment;	high crystal defects
	60–200 °C	universality	
Electrochemical deposition	solution environment;	controllable thickness	nonuniform
	electric field		
Magnetron sputtering deposition	high pressure; electric field	fast deposition speed; low temperature of substrate; controllable thickness	surface roughness; poor uniformity; uncompacted
Chemical vapor deposition	high pressure; high temperature	high crystal quality	high temperature resistant substrate
Sol-gel	solution environment; heat treatment	high purity and uniform; controllable particle size	complex reaction; various variables; long process time
Derive from MOF	solution environment	large surface area	impure

## Data Availability

Not applicable.
